# Crystal structure of 4′-bromo-2,3,5,6-tetra­fluoro­biphenyl-4-carbo­nitrile

**DOI:** 10.1107/S2056989015007847

**Published:** 2015-04-25

**Authors:** Ricarda Heckel, Jürg Hulliger, Anke Schwarzer, Edwin Weber

**Affiliations:** a Institut für Organische Chemie, TU Bergakademie Freiberg, Leipziger Strasse 29, D-09596 Freiberg/Sachsen, Germany; b Department of Chemistry and Biochemistry, University of Berne, Freiestrasse 3, CH-3012 Berne, Switzerland

**Keywords:** crystal structure, biphen­yl, tetra­fluoro substitution, bromo–cyano substitution, π–π^F^ stacking, halogen inter­actions

## Abstract

The title compound, C_13_H_4_BrF_4_N, synthesized from 1,4′-bromo­iodo­benzene and 4-bromo-2,3,5,6-tetra­fluoro­benzo­nitrile in a coupling reaction was found to crystallize in the ortho­rhom­bic space group *P*2_1_2_1_2_1_. The two phenyl rings are rotated with respect to each other by 40.6 (6)°. The mol­ecules inter­act *via* aryl–perfluoroaryl stacking [3.796 (2) and 3.773 (2) Å], resulting in inter­molecular chains along the *a-*axis direction. C—H⋯F contacts of about 2.45 Å connect these chains. In contrast to the structure of the parent compound 4′-bromo­biphenyl-4-carbo­nitrile, CN⋯Br contacts that could have given rise to a linear arrangement of the biphenyl mol­ecules desirable for non-linear optical (NLO) materials are not observed in the packing. Instead, several Br⋯F [3.2405 (17) and 3.2777 (18) Å] and F⋯F [2.894 (2) Å] contacts of side-on type II form an inter­molecular network of zigzag chains. The crystal studied was refined as an inversion twin.

## Related literature   

For crystal structures of 4-cyano-4′-halogene substituted bi­phenyls, see: Gleason *et al.* (1991 ▸) for fluorine, Kronebusch *et al.* (1976 ▸) for bromine, Britton & Gleason (1991 ▸) for iodine. For halogen inter­actions in mol­ecular crystal structures, see: Ramasubbu *et al.* (1986 ▸), Awwadi *et al.* (2006 ▸), Brammer *et al.* (2001 ▸) and Metrangolo *et al.* (2008 ▸). For inter­actions of halogens with cyano groups, see: Desiraju & Harlow (1989 ▸), Süss *et al.* (2005 ▸) and Mukherjee *et al.* (2014 ▸). For fluorine involved into these inter­actions, see: Schwarzer *et al.* (2010 ▸), Merz & Vasylyeva (2010 ▸), Schwarzer & Weber (2008 ▸) and Reichenbächer *et al.* (2005 ▸).
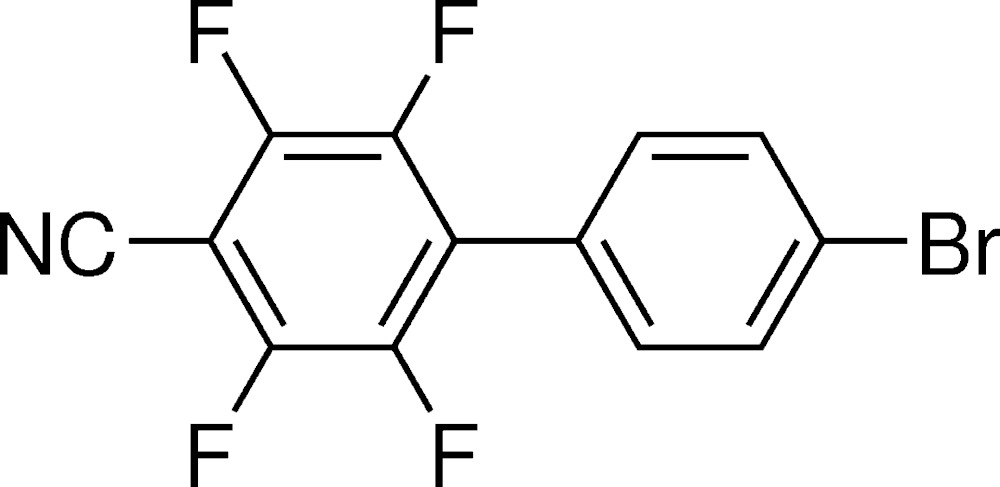


## Experimental   

### Crystal data  

C_13_H_4_BrF_4_N
*M* *_r_* = 330.08
Orthorhombic, 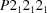

*a* = 7.3560 (15) Å
*b* = 12.107 (2) Å
*c* = 12.723 (3) Å
*V* = 1133.1 (4) Å^3^
*Z* = 4
Mo *K*α radiation
μ = 3.66 mm^−1^
*T* = 93 K
0.49 × 0.13 × 0.10 mm

### Data collection  

Bruker SMART CCD area-detector diffractometer
Absorption correction: multi-scan (*SADABS*; Bruker, 2012) *T*_min_ = 0.486, *T*_max_ = 0.718
18347 measured reflections
3234 independent reflections
2930 reflections with *I* > 2σ(*I*)
*R*_int_ = 0.053

### Refinement  

*R*[*F* ^2^ > 2σ(*F* ^2^)] = 0.028
*wR*(*F* ^2^) = 0.052
*S* = 0.99
3234 reflections
173 parameters
H-atom parameters constrained
Δρ_max_ = 0.46 e Å^−3^
Δρ_min_ = −0.31 e Å^−3^
Absolute structure: refined as an inversion twin.
Absolute structure parameter: 0.011 (9)

### 

Data collection: *SMART* (Bruker, 2007 ▸); cell refinement: *SAINT* (Bruker, 2007 ▸); data reduction: *SAINT*; program(s) used to solve structure: *SHELXTL* (Sheldrick, 2008 ▸); program(s) used to refine structure: *SHELXL2012* (Sheldrick, 2015 ▸); molecular graphics: *XP* (Sheldrick, 2008 ▸); software used to prepare material for publication: *WinGX* (Farrugia, 2012 ▸), *publCIF* (Westrip, 2010 ▸) and *SHELXLE* (Hübschle *et al.*, 2011 ▸).

## Supplementary Material

Crystal structure: contains datablock(s) I, Global. DOI: 10.1107/S2056989015007847/im2464sup1.cif

Structure factors: contains datablock(s) I. DOI: 10.1107/S2056989015007847/im2464Isup2.hkl

Supporting information file. DOI: 10.1107/S2056989015007847/im2464Isup3.cml

**Fig. 1.**
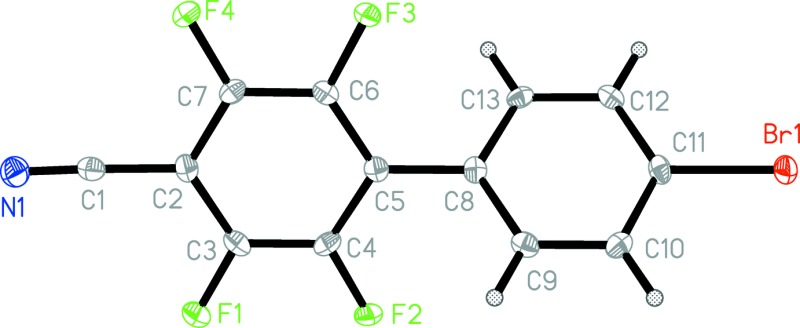
The molecular structure of the title molecule including atom labelling. Displacement ellipsoids drawn at the 50% probability level.

. DOI: 10.1107/S2056989015007847/im2464fig1.tif
The mol­ecular structure of the title mol­ecule including atom labelling. Displacement ellipsoids drawn at the 50% probability level.

**Fig. 2.**
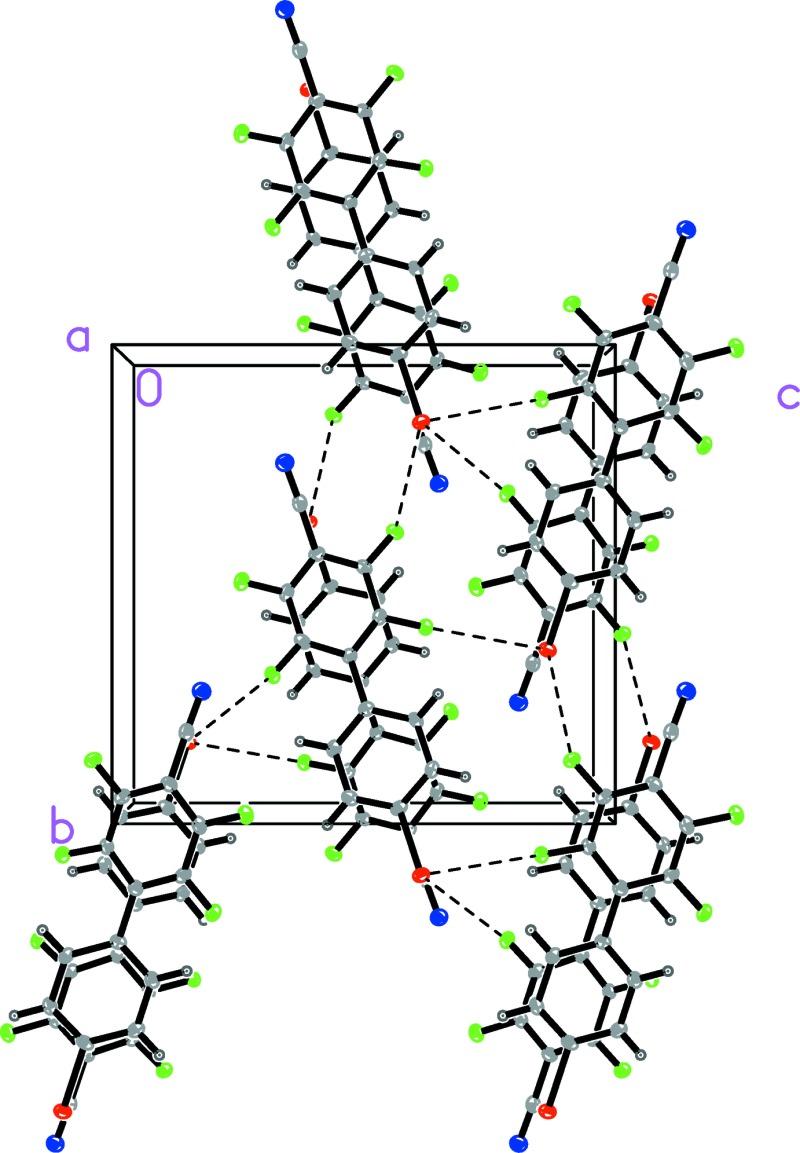
The crystal packing of the title compound showing the stacking interactions along [100].

. DOI: 10.1107/S2056989015007847/im2464fig2.tif
The crystal packing of the title compound showing the stacking inter­actions along [100].

CCDC reference: 1060721

Additional supporting information:  crystallographic information; 3D view; checkCIF report

## Figures and Tables

**Table 1:** Hydrogen-bond geometry (, )

*D*H*A*	*D*H	H*A*	*D* *A*	*D*H*A*
C9H9F2	0.95	2.47	2.882(4)	106
C13H13F3	0.95	2.45	2.865(3)	107

## supplementary crystallographic information

### S1. Synthesis and crystallization

Under inert conditions, 1-bromo-4-iodo­benzene (22.6 g, 80 mmol) in THF (90 mL) was added dropwise to magnesium shaving (1.8 g, 75 mmol. The reaction mixture was refluxed for 90 min. After cooling to room temperature, CuBr (25.8 g, 180 mmol) was added and the mixture was stirred for 1 h at this temperature. Then 15 ml of 1,4-dioxane was added and the mixture was stirred for an hour followed by dropwise addition of a solution of 4-bromo-2,3,5,6-tetra­fluoro­benzo­nitrile (6.4 g, 25 mmol) in toluene (50 ml). After refluxing for 2 d, the mixture was cooled to room temperature, filtered over Celite and freed from solvents removed under reduced pressure. The residue was dissolved in toluene and washed with 3M HCl followed by aqueous NaOH solution. The organic phases were collected, dried over Na_2_SO_4_ and evaporated. The raw product was purified by column chromatography (SiO_2_; eluent: CH_2_Cl_2_/*n*-hexane, 2/1 v/v to yield 1.00 g (12 %) of the title compound. Single crystals suitable for X-ray diffraction were obtained from acetone solution at room temperature. Data for (I): M.p. 133-134 °C. ^1^H NMR(400 MHz; acetone-d_6_): δ_H_ = 7.57 (d, ^3^*J*_HH_ = 8.9, 2H, H-9, H-13), 7.82 (d, ^3^*J*_HH_ = 8.9, 2H, H-10, H12) ppm. ^13^C NMR (100 MHz; acetone-d_6_): δ_C_ = 94.30 (d, ^2^*J*_CF_ = 17.4, C-2), 108.62 (t, ^3^*J*_CF_ = -3.7, C-1), 125.52, 126.32 (s, C-8, C11), 127.04 (t, ^2^*J*_CF_ = 17.4, C-5), 133.05 (t, ^4^*J*_CF_ = 2.5, C-9), 133.32 (s, C-10), 143.39, 146.69 (d, ^1^*J*_CF_ = -147.2, C-4), 147.01, 150.43 (d, ^1^*J*_CF_ = -265.5, C-3) ppm. ^19^F NMR(376 MHz; acetone-d_6_): δ_F_ = -136.15 (F-1, d, ^3^*J*_FF_ = 9.3 ), -142.53 (F-2, d, ^3^*J*_FF_ = 9.3) ppm. GC—MS (m/z) 329 [M]^+^, 250 [M—Br]^+^, 231 [-F]^+^, 200 [-CF]^+^, 125, 99, 74, 50.

### S2. Refinement details

The C-bound H atoms were positioned geometrically and allowed to ride on their parent atoms: C–H = 0.95 Å for aryl H atoms, with [*U*_iso_(H) = 1.2*U*_eq_(C)].

### Crystal data

**Table:**

C_13_H_4_BrF_4_N	*D*_x_ = 1.935 Mg m^−^^3^
*M**_r_* = 330.08	Mo *K*α radiation, λ = 0.71073 Å
Orthorhombic, *P*2_1_2_1_2_1_	Cell parameters from 4750 reflections
*a* = 7.3560 (15) Å	θ = 3.2–28.4°
*b* = 12.107 (2) Å	µ = 3.66 mm^−^^1^
*c* = 12.723 (3) Å	*T* = 93 K
*V* = 1133.1 (4) Å^3^	Splitter, colorless
*Z* = 4	0.49 × 0.13 × 0.10 mm
*F*(000) = 640	

### Data collection

**Table:**

Bruker SMART CCD area-detector diffractometer	2930 reflections with *I* > 2σ(*I*)
Radiation source: fine-focus sealed tube	*R*_int_ = 0.053
phi and ω scans	θ_max_ = 29.8°, θ_min_ = 2.3°
Absorption correction: multi-scan (*SADABS*; Bruker, 2012)	*h* = −10→10
*T*_min_ = 0.486, *T*_max_ = 0.718	*k* = −16→16
18347 measured reflections	*l* = −17→17
3234 independent reflections	

### Refinement

**Table:**

Refinement on *F*^2^	Hydrogen site location: inferred from neighbouring sites
Least-squares matrix: full	H-atom parameters constrained
*R*[*F*^2^ > 2σ(*F*^2^)] = 0.028	*w* = 1/[σ^2^(*F*_o_^2^) + (0.0144*P*)^2^] where *P* = (*F*_o_^2^ + 2*F*_c_^2^)/3
*wR*(*F*^2^) = 0.052	(Δ/σ)_max_ = 0.001
*S* = 0.99	Δρ_max_ = 0.46 e Å^−^^3^
3234 reflections	Δρ_min_ = −0.31 e Å^−^^3^
173 parameters	Absolute structure: Refined as an inversion twin.
0 restraints	Absolute structure parameter: 0.011 (9)

### Special details

**Table:**

Geometry. All e.s.d.'s (except the e.s.d. in the dihedral angle between two l.s. planes) are estimated using the full covariance matrix. The cell e.s.d.'s are taken into account individually in the estimation of e.s.d.'s in distances, angles and torsion angles; correlations between e.s.d.'s in cell parameters are only used when they are defined by crystal symmetry. An approximate (isotropic) treatment of cell e.s.d.'s is used for estimating e.s.d.'s involving l.s. planes.
Refinement. Refined as a 2-component inversion twin.

### Fractional atomic coordinates and isotropic or equivalent isotropic displacement parameters (Å^2^)

**Table:**

	*x*	*y*	*z*	*U*_iso_*/*U*_eq_	
Br1	0.89811 (4)	0.35795 (3)	0.37922 (2)	0.01853 (8)	
F1	0.7744 (2)	1.00596 (16)	0.75920 (12)	0.0196 (4)	
F2	0.7639 (2)	0.79698 (15)	0.69387 (12)	0.0179 (4)	
F3	1.0359 (2)	0.90632 (14)	0.37137 (13)	0.0163 (4)	
F4	1.0323 (2)	1.11450 (14)	0.43493 (13)	0.0196 (4)	
N1	0.9005 (4)	1.2681 (2)	0.66563 (19)	0.0208 (6)	
C1	0.9026 (4)	1.1790 (2)	0.6343 (2)	0.0161 (6)	
C2	0.9029 (5)	1.0664 (2)	0.5984 (2)	0.0153 (6)	
C3	0.8371 (4)	0.9812 (3)	0.6627 (2)	0.0154 (7)	
C4	0.8354 (4)	0.8729 (3)	0.6295 (2)	0.0147 (6)	
C5	0.9004 (4)	0.8418 (2)	0.5296 (2)	0.0131 (6)	
C6	0.9650 (4)	0.9283 (3)	0.4667 (2)	0.0129 (6)	
C7	0.9665 (4)	1.0369 (3)	0.4992 (2)	0.0144 (6)	
C8	0.8998 (5)	0.7249 (2)	0.4938 (2)	0.0133 (6)	
C9	0.9476 (4)	0.6389 (3)	0.5620 (2)	0.0145 (6)	
H9	0.9807	0.6555	0.6324	0.017*	
C10	0.9474 (4)	0.5302 (3)	0.5286 (2)	0.0161 (7)	
H10	0.9795	0.4725	0.5756	0.019*	
C11	0.8997 (5)	0.5065 (2)	0.4254 (2)	0.0148 (6)	
C12	0.8517 (4)	0.5894 (3)	0.3554 (2)	0.0153 (7)	
H12	0.8191	0.5720	0.2851	0.018*	
C13	0.8520 (4)	0.6986 (3)	0.3898 (2)	0.0142 (6)	
H13	0.8196	0.7560	0.3424	0.017*	

### Atomic displacement parameters (Å^2^)

**Table:**

	*U*^11^	*U*^22^	*U*^33^	*U*^12^	*U*^13^	*U*^23^
Br1	0.02368 (15)	0.01300 (14)	0.01890 (13)	−0.00129 (14)	0.00259 (14)	−0.00242 (13)
F1	0.0229 (10)	0.0218 (10)	0.0141 (8)	0.0003 (8)	0.0031 (7)	−0.0022 (7)
F2	0.0213 (10)	0.0185 (10)	0.0138 (8)	−0.0028 (8)	0.0029 (7)	0.0034 (7)
F3	0.0201 (9)	0.0163 (9)	0.0125 (7)	0.0014 (7)	0.0034 (7)	−0.0004 (8)
F4	0.0265 (10)	0.0143 (10)	0.0179 (8)	−0.0015 (7)	0.0042 (7)	0.0030 (7)
N1	0.0216 (14)	0.0193 (15)	0.0215 (12)	0.0015 (14)	0.0011 (13)	−0.0013 (11)
C1	0.0153 (14)	0.0200 (15)	0.0130 (13)	−0.0011 (13)	0.0005 (14)	0.0010 (11)
C2	0.0156 (13)	0.0137 (14)	0.0165 (13)	0.0012 (14)	−0.0020 (13)	−0.0026 (11)
C3	0.0133 (15)	0.0204 (18)	0.0125 (13)	0.0020 (13)	−0.0003 (11)	−0.0034 (12)
C4	0.0121 (13)	0.0149 (16)	0.0169 (13)	−0.0017 (11)	−0.0013 (12)	0.0049 (14)
C5	0.0112 (12)	0.0144 (15)	0.0136 (11)	0.0014 (14)	−0.0040 (12)	−0.0006 (11)
C6	0.0110 (14)	0.0162 (16)	0.0116 (13)	0.0024 (12)	−0.0007 (11)	−0.0012 (12)
C7	0.0136 (14)	0.0138 (16)	0.0160 (14)	0.0003 (12)	−0.0008 (11)	0.0035 (12)
C8	0.0101 (13)	0.0134 (15)	0.0164 (12)	−0.0001 (13)	0.0009 (13)	−0.0013 (11)
C9	0.0117 (14)	0.0187 (16)	0.0133 (12)	−0.0030 (13)	−0.0021 (10)	−0.0017 (14)
C10	0.0155 (17)	0.0149 (16)	0.0178 (14)	0.0010 (12)	0.0008 (12)	0.0033 (13)
C11	0.0125 (13)	0.0136 (15)	0.0183 (13)	−0.0029 (14)	0.0017 (14)	−0.0048 (11)
C12	0.0147 (16)	0.0191 (17)	0.0120 (14)	−0.0006 (12)	0.0002 (10)	−0.0025 (12)
C13	0.0136 (15)	0.0151 (15)	0.0137 (13)	0.0018 (11)	−0.0005 (11)	0.0045 (12)

### Geometric parameters (Å, º)

**Table:**

Br1—C11	1.892 (3)	C5—C8	1.487 (4)
F1—C3	1.346 (3)	C6—C7	1.379 (4)
F2—C4	1.339 (3)	C8—C9	1.400 (4)
F3—C6	1.347 (3)	C8—C13	1.406 (4)
F4—C7	1.336 (3)	C9—C10	1.383 (4)
N1—C1	1.150 (4)	C9—H9	0.9500
C1—C2	1.438 (4)	C10—C11	1.389 (4)
C2—C7	1.393 (4)	C10—H10	0.9500
C2—C3	1.402 (4)	C11—C12	1.388 (4)
C3—C4	1.377 (4)	C12—C13	1.392 (4)
C4—C5	1.409 (4)	C12—H12	0.9500
C5—C6	1.401 (4)	C13—H13	0.9500
			
N1—C1—C2	178.1 (3)	C9—C8—C13	118.6 (3)
C7—C2—C3	117.1 (3)	C9—C8—C5	121.2 (2)
C7—C2—C1	122.1 (3)	C13—C8—C5	120.3 (3)
C3—C2—C1	120.8 (2)	C10—C9—C8	121.1 (3)
F1—C3—C4	119.3 (3)	C10—C9—H9	119.4
F1—C3—C2	119.1 (3)	C8—C9—H9	119.4
C4—C3—C2	121.6 (3)	C9—C10—C11	119.2 (3)
F2—C4—C3	118.0 (3)	C9—C10—H10	120.4
F2—C4—C5	120.1 (3)	C11—C10—H10	120.4
C3—C4—C5	121.9 (3)	C12—C11—C10	121.4 (3)
C6—C5—C4	115.5 (3)	C12—C11—Br1	119.1 (2)
C6—C5—C8	122.5 (2)	C10—C11—Br1	119.5 (2)
C4—C5—C8	122.0 (3)	C11—C12—C13	119.0 (3)
F3—C6—C7	117.1 (3)	C11—C12—H12	120.5
F3—C6—C5	119.8 (3)	C13—C12—H12	120.5
C7—C6—C5	123.0 (3)	C12—C13—C8	120.7 (3)
F4—C7—C6	119.3 (3)	C12—C13—H13	119.6
F4—C7—C2	119.8 (3)	C8—C13—H13	119.6
C6—C7—C2	120.9 (3)		
			
C7—C2—C3—F1	179.9 (3)	C5—C6—C7—C2	0.2 (5)
C1—C2—C3—F1	−0.5 (4)	C3—C2—C7—F4	−179.4 (3)
C7—C2—C3—C4	0.1 (5)	C1—C2—C7—F4	1.0 (5)
C1—C2—C3—C4	179.7 (3)	C3—C2—C7—C6	−0.3 (4)
F1—C3—C4—F2	2.6 (4)	C1—C2—C7—C6	−179.9 (3)
C2—C3—C4—F2	−177.5 (3)	C6—C5—C8—C9	139.2 (3)
F1—C3—C4—C5	−179.5 (3)	C4—C5—C8—C9	−40.8 (4)
C2—C3—C4—C5	0.3 (5)	C6—C5—C8—C13	−40.4 (5)
F2—C4—C5—C6	177.3 (3)	C4—C5—C8—C13	139.6 (3)
C3—C4—C5—C6	−0.5 (4)	C13—C8—C9—C10	−0.3 (4)
F2—C4—C5—C8	−2.6 (4)	C5—C8—C9—C10	−179.9 (3)
C3—C4—C5—C8	179.6 (3)	C8—C9—C10—C11	0.3 (4)
C4—C5—C6—F3	177.6 (2)	C9—C10—C11—C12	−0.2 (5)
C8—C5—C6—F3	−2.4 (4)	C9—C10—C11—Br1	−179.8 (2)
C4—C5—C6—C7	0.2 (4)	C10—C11—C12—C13	0.1 (5)
C8—C5—C6—C7	−179.8 (3)	Br1—C11—C12—C13	179.7 (2)
F3—C6—C7—F4	1.8 (4)	C11—C12—C13—C8	−0.1 (4)
C5—C6—C7—F4	179.3 (3)	C9—C8—C13—C12	0.2 (4)
F3—C6—C7—C2	−177.3 (3)	C5—C8—C13—C12	179.8 (3)

### Hydrogen-bond geometry (Å, º)

**Table:**

*D*—H···*A*	*D*—H	H···*A*	*D*···*A*	*D*—H···*A*
C9—H9···F2	0.95	2.47	2.882 (4)	106
C13—H13···F3	0.95	2.45	2.865 (3)	107
