# Supplementary figures and images for: Crystal structure of 4′-bromo-2,3,5,6-tetra­fluoro­biphenyl-4-carbo­nitrile
Source: Acta Crystallogr E Crystallogr Commun. 2015 Apr 25;71(Pt 5):o347–8. doi: 10.1107/S2056989015007847 (PMC4420037; doi:10.1107/S2056989015007847)

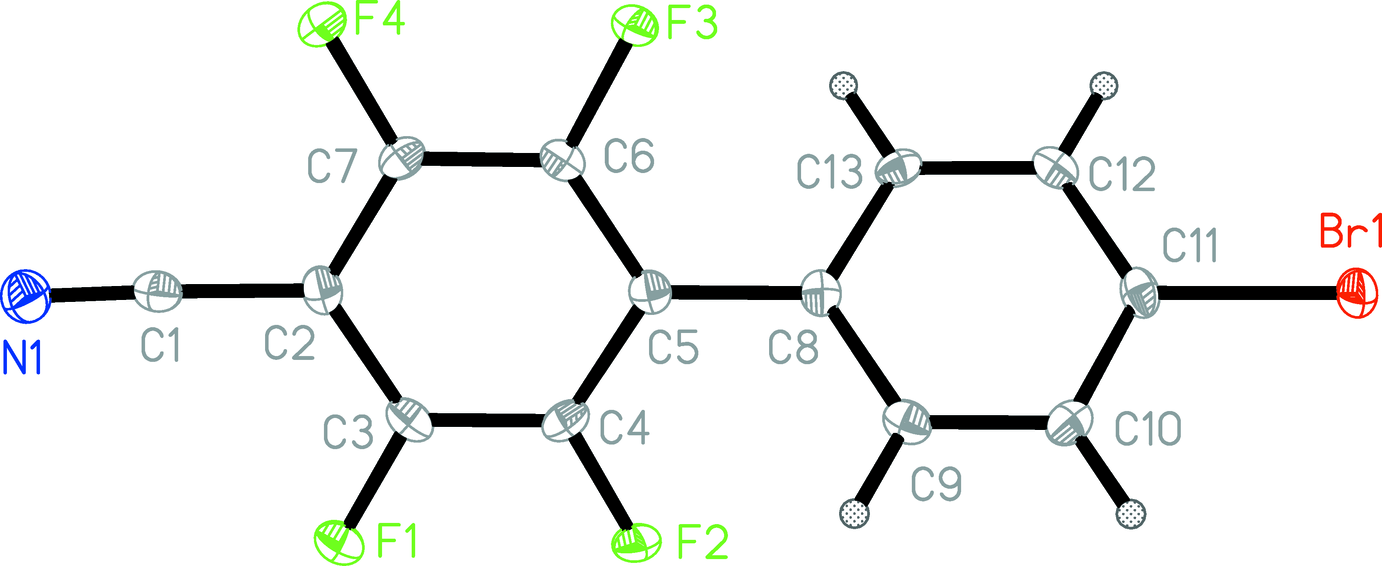

Supplement: Supplementary file 4 [file e-71-0o347-fig1.tif]

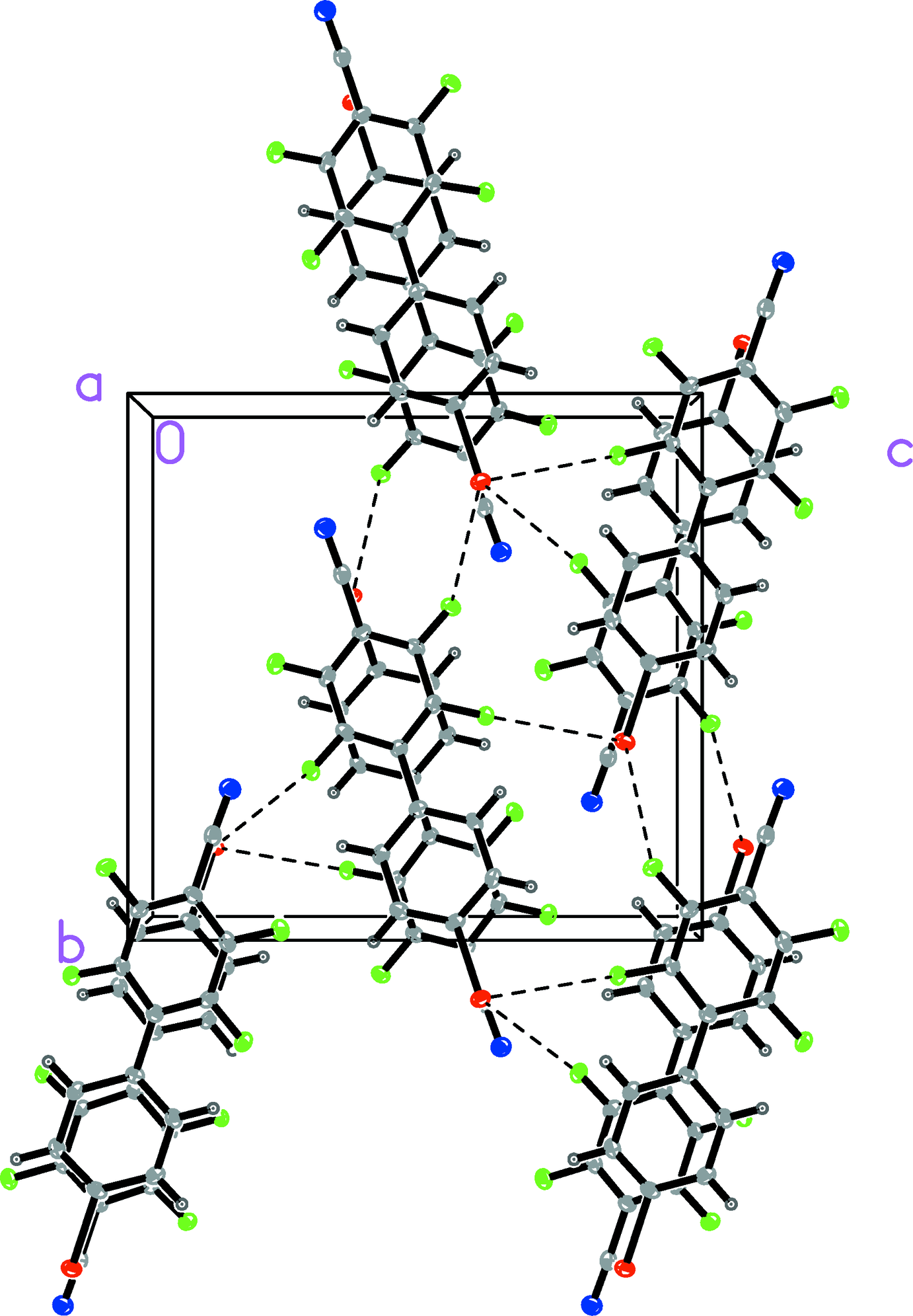

Supplement: Supplementary file 5 [file e-71-0o347-fig2.tif]
